# Neuroanatomy of mud dragons: a comprehensive view of the nervous system in *Echinoderes* (Kinorhyncha) by confocal laser scanning microscopy

**DOI:** 10.1186/s12862-019-1405-4

**Published:** 2019-04-08

**Authors:** María Herranz, Brian S. Leander, Fernando Pardos, Michael J. Boyle

**Affiliations:** 1 Departments of Zoology and Botany, University of British Columbia. Biodiversity Research Centre, 2212 Main Mall, Vancouver, BC V6T 1Z4 Canada; 2 Departamento de Biodiversidad, Ecología y Evolución, Universidad Complutense de Madrid, C/José Antonio Novais, 22040 Madrid, Spain; 3 Smithsonian Institution, Smithsonian Marine Station at Fort Pierce, 701 Seaway Drive, Fort Pierce, Florida, 34949 USA

**Keywords:** Ecdysozoa, Nervous system, Nerve cord, Morphology, Scalidophora, Segmentation

## Abstract

**Background:**

The Scalidophora (Kinorhyncha, Loricifera and Priapulida) have an important phylogenetic position as early branching ecdysozoans, yet the architecture of their nervous organ systems is notably underinvestigated. Without such information, and in the absence of a stable phylogenetic context, we are inhibited from producing adequate hypotheses about the evolution and diversification of ecdysozoan nervous systems. Here, we utilize confocal laser scanning microscopy to characterize serotonergic, tubulinergic and FMRFamidergic immunoreactivity patterns in a comparative neuroanatomical study with three species of *Echinoderes*, the most speciose, abundant and diverse genus within Kinorhyncha.

**Results:**

Neuroanatomy in *Echinoderes* as revealed by acetylated α-tubulin immunoreactivity includes a circumpharyngeal brain and ten neurite bundles in the head region that converge into five longitudinal nerves within the trunk. The ventral nerve cord is ganglionated, emerging from the brain with two connectives that converge in trunk segments 2–3, and diverge again within segment 8. The longitudinal nerves and ventral nerve cord are connected by two transverse neurites in segments 2–9. Differences among species correlate with the number, position and innervation of cuticular structures along the body. Patterns of serotoninergic and FMRFamidergic immunoreactivity correlate with the position of the brain neuropil and the ventral nerve cord. Distinct serotonergic and FMRFamidergic somata are associated with the brain neuropil and specific trunk segments along the ventral nerve cord.

**Conclusions:**

Neural architecture is highly conserved across all three species, suggesting that our results reveal a pattern that is common to more than 40% of the species within Kinorhyncha. The nervous system of *Echinoderes* is segmented along most of the trunk; however, posterior trunk segments exhibit modifications that are likely associated with sensorial, motor or reproductive functions. Although all kinorhynchs show some evidence of an externally segmented trunk, it is unclear whether external segmentation matches internal segmentation of nervous and muscular organ systems across Kinorhyncha, as we observed in *Echinoderes*. The neuroanatomical data provided in this study not only expand the limited knowledge on kinorhynch nervous systems but also establish a comparative morphological framework within Scalidophora that will support broader inferences about the evolution of neural architecture among the deepest branching lineages of the Ecdysozoa.

**Electronic supplementary material:**

The online version of this article (10.1186/s12862-019-1405-4) contains supplementary material, which is available to authorized users.

## Background

Nervous organ systems are responsible for receiving, processing and transmitting information to coordinate movement and behavior across the Metazoa [1, 2]. All extant nervous systems reflect different evolutionary pressures on different life histories, resulting in variation of overall organization ranging from nerve nets to highly complex centralized systems [3, 4]. Our current understanding of bilaterian neural architecture is biased towards certain animal groups such as arthropods and vertebrates, which only represent a fraction of the existing diversity of nervous systems. Large gaps of knowledge prevail, especially among most of the “lesser-known phyla” [1]. Studies of these so called lesser-known groups will help us to resolve evolutionary changes among animals that occupy key positions in the tree of life [2]. Such is the case for the Cycloneuralia which includes Scalidophora (Kinorhyncha, Loricifera and Priapulida) and Nematoida (Nematoda and Nematomorpha) [5]. All of these lineages split deep within the molting animals or Ecdysozoa. Thus far the monophyly of Cycloneuralia is supported solely by morphological descriptions associated with the shared structure of their circumpharyngeal ring-like brain [5, 6]. However, a monophyletic Cycloneuralia remains contentious in different phylogenetic analyses that more often show them as paraphyletic (e.g. [7–9]). Critical morphological reassessments are necessary to reconcile current incompatibilities between molecular and morphological datasets.

The monophyly of Scalidophora, basal ecdysozoan groups with a radial head bearing scalids [5], is also uncertain without a stable phylogenetic framework (summarized in [10]). Within Scalidophora, Kinorhyncha and Priapulida typically resolve as sister groups in most phylogenetic investigations [9, 11, 12]; however, Loricifera is rarely included in the datasets analyzed (except for [13–15]). Laumer et al. [16] included transcriptomic data from Loricifera and Priapulida in a recent phylogenetic hypothesis but did not include Kinorhyncha. The results of that study recovered a clade of loriciferans and priapulids, which indicates only partial evidence of scalidophoran monophyly due to the absence of kinorhynchs, a relatively low support for the clade, and a limited taxon sampling within ecdysozoans. More inclusive detailed studies of the nervous system within Scalidophora are therefore required to help assess evolutionary relationships among cycloneuralian taxa.

Despite the paucity of data on the scalidophoran nervous systems, several studies on priapulid morphology are available, which include both macroscopic (*Halicryptus*, *Priapulus*, *Priapulopsis*) [17] and microscopic genera (*Tubiluchus*, *Meiopriapulus*) [18, 19], as well as adult and larval stages [20, 21]. Studies of loriciferans nervous systems are restricted to a few ultrastructural investigations in a couple of species: *Nanaloricus mysticus* and *Pliciloricus enigmaticus* [22, 23], whereas no immunohistochemical data are currently available. Data on the nervous system architecture of kinorhynchs is limited. Except for the detailed and extensive ultrastructure work by Nebelsick with *Echinoderes capitatus* [24, 25], very few studies have been exclusively focused on the nervous system in Kinorhyncha. Most of the available data are from general morphological studies of ultrastructure such as the one by Kristensen and Higgins [26]. These studies showed that the nervous system in Kinorhyncha is composed of a circumpharyngeal brain, divided into anterior somata, neuropil and posterior somata, and several longitudinal nerves in the trunk that are generally connected by two commissures per segment [25–27]. Within *E. capitatus*, the ventral nerve cord is ganglionated and unpaired, arising from “the fusion” of two of the ten longitudinal neurite bundles extending posteriorly from the introvert [25]. More recent studies applied a combination of immunohistochemistry and confocal microscopy in one [28] or multiple species [29]. These immunohistochemical studies have been pivotal to our understanding of kinorhynch neuroanatomy providing information on serotoninergic and tubulinergic components of the central nervous system; however, more comprehensive investigations with additional markers and a broader selection of genera and species are still necessary. Importantly, among the basal ecdysozoan lineages, kinorhynchs are the only group that clearly exhibit morphological segmentation of internal and external structures derived from ectodermal and mesodermal tissues (e.g. cuticle, spines, muscles, nerves) along the anterior-posterior axis [27]. This makes Kinorhyncha a key taxon to guide us toward a deeper understanding of the early evolution of segmentation within Ecdysozoa.

*Echinoderes* is by far the most speciose genus within kinorhynchs, accommodating more than 110 of ca. 270 species, and representing approximately 40% of the known diversity. Appropriately, this study provides for the first time a comprehensive neuroanatomical description of three species that exemplify the morphological variability within the *Echinoderes* genus with contrasting differences in the configuration of their cuticular characters including spines, tubes, sieve plates, terminal spines and sensory spots (see [30–32]): *Echinoderes horni* Higgins, 1983; *Echinoderes spinifurca* Sørensen et al., 2005 and *Echinoderes ohtsukai* Yamasaki and Kajihara, 2012. In each species, we analyzed serotonergic, tubulinergic and FMRFamidergic immunoreactivity patterns along with markers for musculature (phalloidin) and DNA (propidium iodide) as positional landmarks of anatomy. Our investigation, which includes 3D reconstruction of those patterns, serves to complement previous ultrastructural studies with new observations and interpretations, thus increasing the availability of detailed and more complete neuroanatomical data within Kinorhyncha. The results of this study will enable a broader comprehension of internal organization and help to establish a comparative framework among closely related scalidophoran lineages. Furthermore, our data will serve as a reference for better understanding the evolution of nervous systems across Ecdysozoa.

## Results

### External morphology of *Echinoderes*

**Fig. 1:**
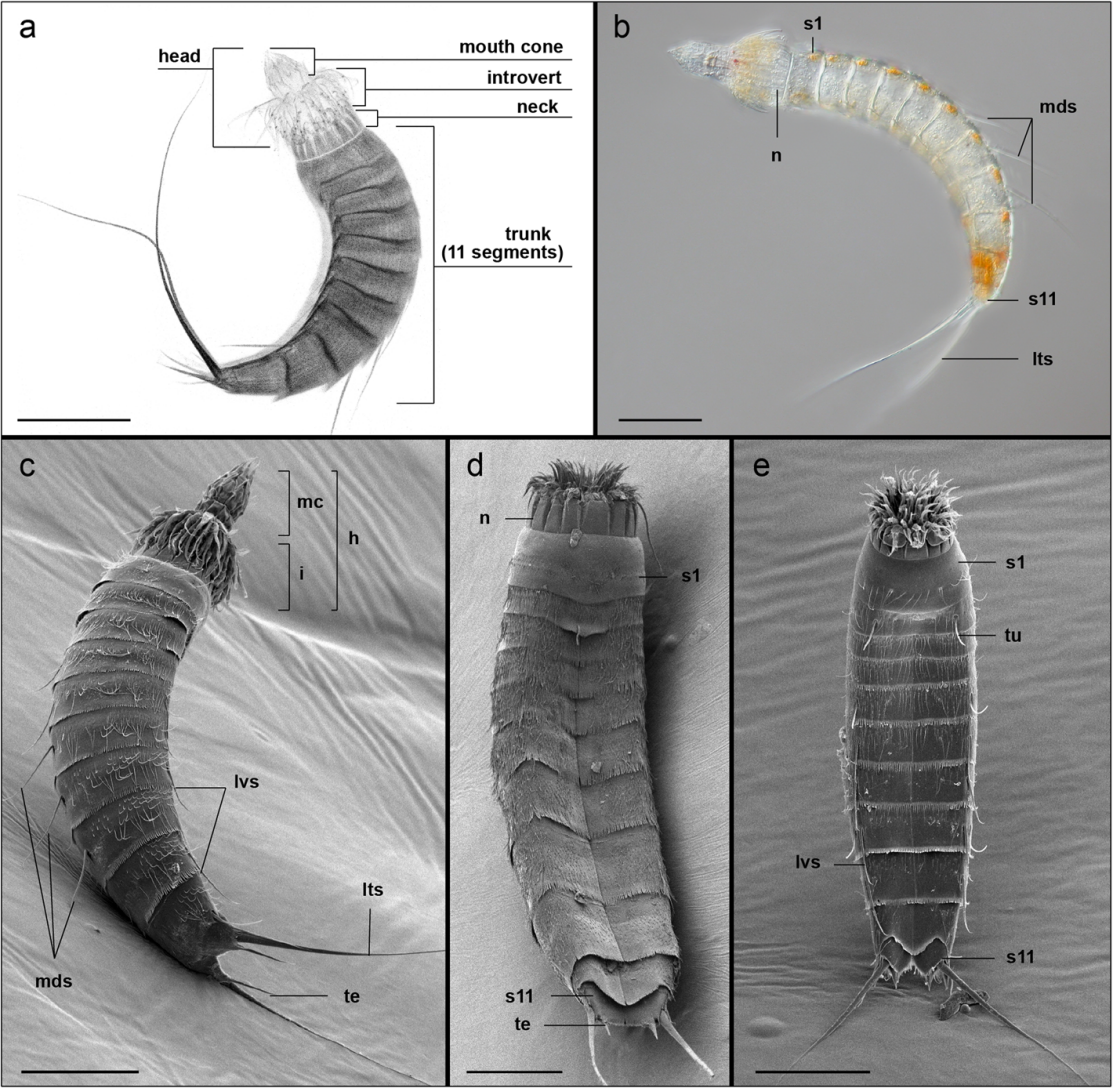
External anatomy and body plan organization of *Echinoderes* (Kinorhyncha). All views with anterior to the top. **a** Confocal z-stack projection of the autofluorescent cuticle of *Echinoderes spinifurca*, lateral view with ventral to the left, head extended. **b** Differential interference contrast (DIC) micrograph of *E. spinifurca*, lateral view with ventral to the left, head extended. **c-e** Scanning electron micrographs (SEM) of *E. spinifurca* in right dorsolateral view, head extended (**c**), *Echinoderes ohtsukai* in ventral view, head retracted (**d**), and *Echinoderes horni* in ventral view, head partially retracted (**e**). Scale bars: 50 μm in all panels. Abbreviations: h, head; i, introvert; lts, lateral terminal spine; lvs, lateroventral spine; mc, mouth cone; mds, middorsal spine; n, neck; s1, segment 1; s11, segment 11; te, tergal extension; tu, tube

Anatomically, *Echinoderes* species can be divided from anterior to posterior in three distinct regions including a radially symmetric head and neck, and a bilaterally symmetric trunk (Fig. 1a). The head is composed of an eversible introvert and a protrusible mouth cone. The introvert carries several rows of cuticular spines or scalids that are radially arranged around the mouth cone. The mouth cone bears a ring of nine articulated outer oral styles, typically very prominent, and three rings of smaller, non-articulated inner oral styles that encircle the mouth. The neck region is composed of 16 radially-arranged cuticular plates, known as placids, that function as a closing system when the head is retracted within the trunk (Fig. 1a, b, d). The trunk is divided into 11 segments that vary in length and articulate with each other (Fig. 1). The first two segments are ring-like whereas segments 3–11 are divided into one tergal (dorsal) and two sternal (ventral) plates. The trunk can bear acicular spines that vary among species in their appearance, size and position (Fig. 1b-c). The last trunk segment consistently exhibits a pair of lateral terminal spines. Furthermore, males show three pairs of penile spines whereas females show a single pair of lateral accessory spines. Differences in the external morphology among *Echinoderes* species are associated with the presence, size, number and position of spines and other cuticular structures including sensory spots, tubes, hairs and glands as well as trunk proportions. We investigated the nervous system in the following three kinorhynch species from the genus *Echinoderes: E. horni, E. spinifurca* and *E. ohtsukai* (Fig. 1)*.*

### Tubulinergic nervous system

**Fig. 2:**
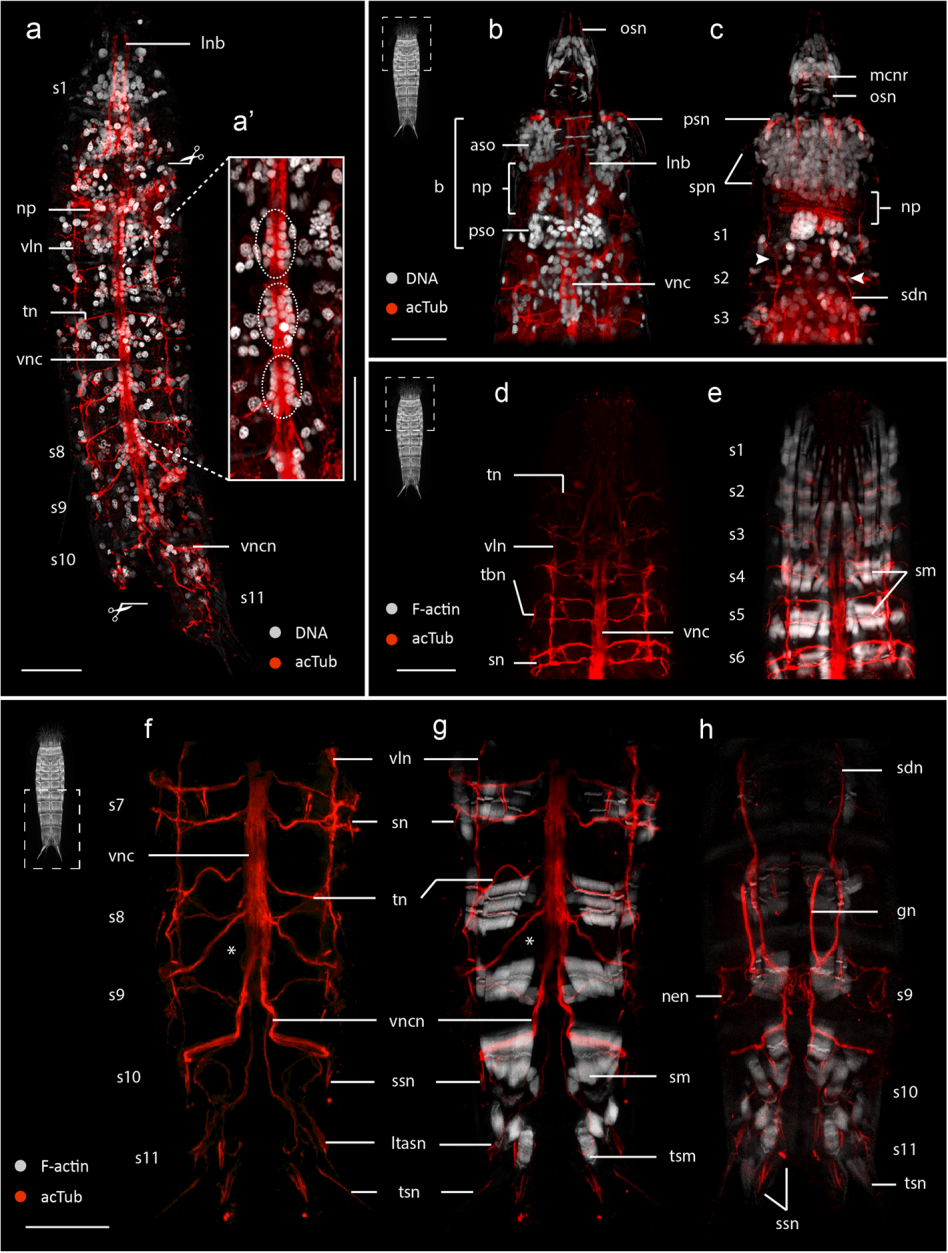
Acetylated α-tubulin-LIR in the nervous system of *Echinoderes*. a-h Confocal z-stack projections of specimens co-labeled for acTub-LIR and DNA (**a-c**) or F-actin (**e, g-h**). Anterior is up in all panels. Specimen silhouettes with dashed squares (**b-h**) indicate the axial region of each z-stack. **a**-**c** *Echinoderes spinifurca*. **d**-**h** *Echinoderes horni*. **a** Ventral view with head retracted showing the ventral nerve cord, two longitudinal ventrolateral nerves and transverse neurites within segments. **a**’ Magnified view of the ventral nerve cord showing clusters of cell nuclei within segmental ganglia (dashed circles). **b-c** Anterior region with introvert and mouth cone everted in (**b**) ventral and (**c**) dorsal views. The brain region is divided into aggregations of anterior and posterior somata with a centralized neuropil. **c** The longitudinal neurites fuse (arrowheads) in segment 1. **d-e** Segments 1–6 with head retracted. **e** Same image as in (**d**) with musculature (F-actin). **f-h** Segments 7–11 in (**f**-**g**) ventral and (**h**) dorsal views. **g** Same image as in (**f**) with musculature (F-actin). Scissor symbols mark the positions of cuticle dissection to facilitate antibody penetration. An asterisk (**f**-**g**) marks the location where the ventral nerve cord divides into left and right branches. Scale bars: 20 μm. Abbreviations: aso, anterior somata; b, brain; nen, nephridial neurite; np, neuropil; lnb, longitudinal neurite bundle; ltasn, lateral terminal accessory spine neurite; mcnr, mouth cone nerve ring; gn, gonad neurite; osn, oral style neurite; psn, primary spinoscalids neurite; pso, posterior somata; s1–11, trunk segment number; sdn, subdorsal longitudinal nerve; sm, segmental muscles; sn, spine neurite; spn, spinoscalid neurite; ssn, sensory spot neurite; tbn, tube neurite; tn, transverse neurite; tsm, terminal spine muscle; tsn, terminal spine neurite; vln, ventrolateral nerve; vnc, ventral nerve cord; vncn, ventral nerve cord neurite


Acetylated α-tubulin-like immunoreactivity (acTub-LIR) was detected within *E. spinifurca* (22 specimens), *E. horni* (21 specimens) (Additional files 1 and 2) and *E. ohtsukai* (14 specimens) (Figs. 2, 4, 5 and 6). Intraspecific variation of acTub-LIR was not observed among specimens. All three species exhibit highly similar architecture of the tubulinergic nervous system with minor variations between them. Interspecific variation of acTub-LIR was primarily associated with innervation of different cuticular structures such as sensory spots, spines, tubes and nephridiopores. Variability in the presence, absence, distribution and number of cuticular structures among these species are considered species-specific diagnostic characters.

**Additional file 2:** Three-dimensional reconstruction of the tubulinergic nervous system (acetylated α-tubulin-LIR) of segments 6–11 in *Echinoderes horni.* Movie showing 3D reconstruction of acTub-LIR. This specimen rotates 360° about the anterior-posterior and left-right axes; anterior is to the top. Musculature (F-actin) is shown in gray. Color code: red, ventral nerve cord and associated posterior peripheral neurites; yellow, transverse neurites; light green, ventrolateral longitudinal nerves; dark blue, subdorsal longitudinal nerves; light blue, cuticular spine neurites; magenta, nephridial neurites; pink, sensory spot neurites. (MP4 6980 kb)


**Fig. 3:**
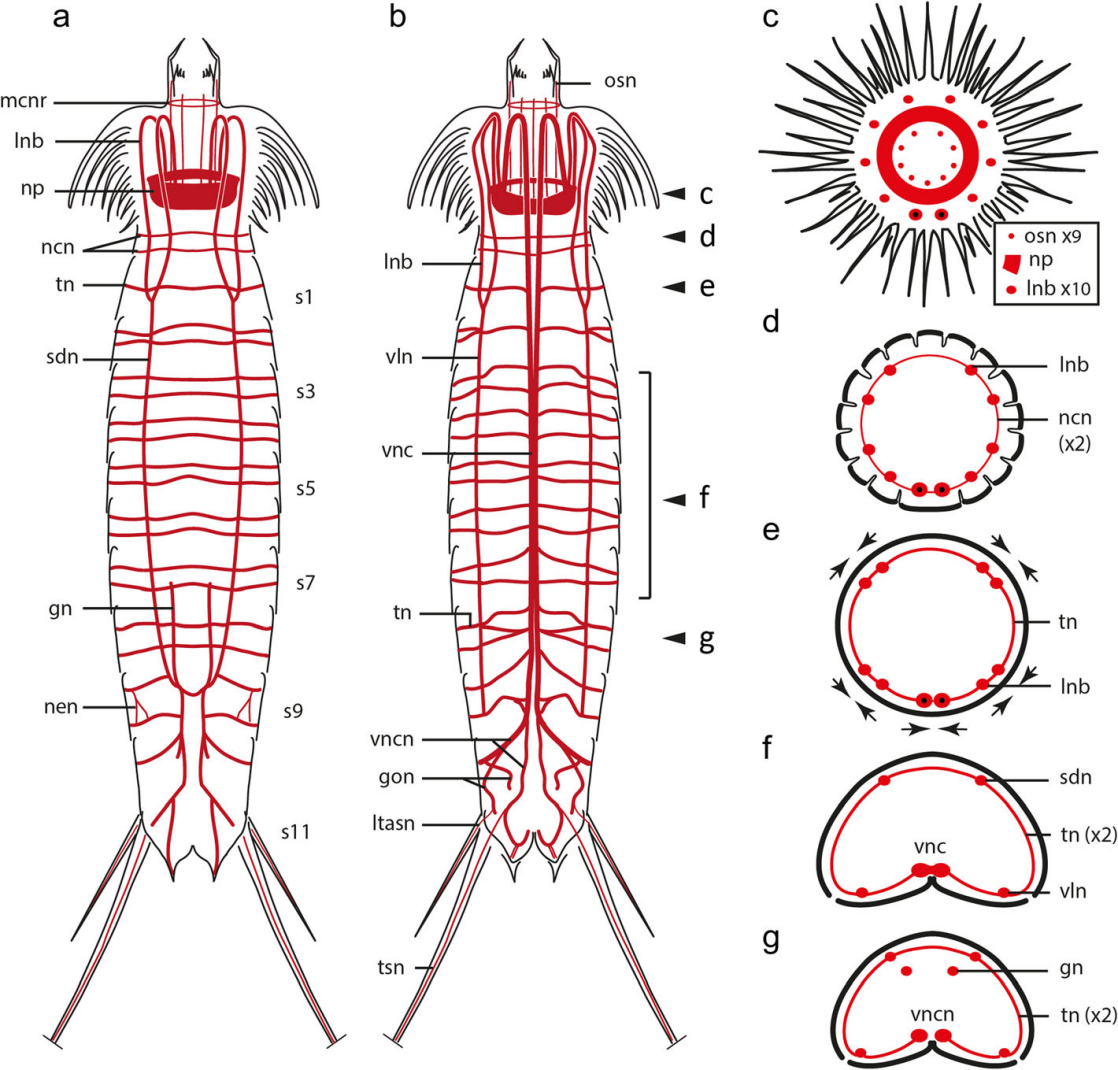
Schematic representation of acetylated α-tubulin-LIR in *Echinoderes*. Only conserved traits across species are represented. For clarity, innervation and external arrangements of species-specific cuticular characters and head scalids have been omitted. Anterior is up in (**a-b**), dorsal is up in (**c-g**). **a-b** Overview of tubulinergic elements of the nervous system in (**a**) dorsal and (**b**) ventral views. Arrowheads in (**b**) mark the anterior-posterior axial positions of cross-section schematics in (**c-g)**. **c** Introvert. Note the outer ring has 10 radially arranged longitudinal neurite bundles (× 10), the middle ring corresponds with the neuropil, and the inner ring shows 9 oral styles neurites (× 9), without the middorsal neurite (see legend). **d** Neck. **e** Segment 1 (arrows mark convergent longitudinal bundles). The two ventral bundles with black dots in (**c-e)** mark the unfused condition of the ventral nerve cord. **f** Segments 3–7. **g** Segment 8. Abbreviations: gn, gonad neurite; gon, gonopore neurite; lnb, longitudinal neurite bundle; ltasn, lateral terminal accessory spine neurite; mcnr, mouth cone nerve ring; ncn, neck circular neurite; nen, nephridial neurite; np, neuropil; osn, oral styles neurite; sdn, subdorsal longitudinal nerve; s1–11, trunk segment number; tn, transverse neurite; tsn, terminal spine neurite; vln, ventrolateral nerve; vnc, ventral nerve cord; vncn, ventral nerve cord neurite

**Fig. 4:**
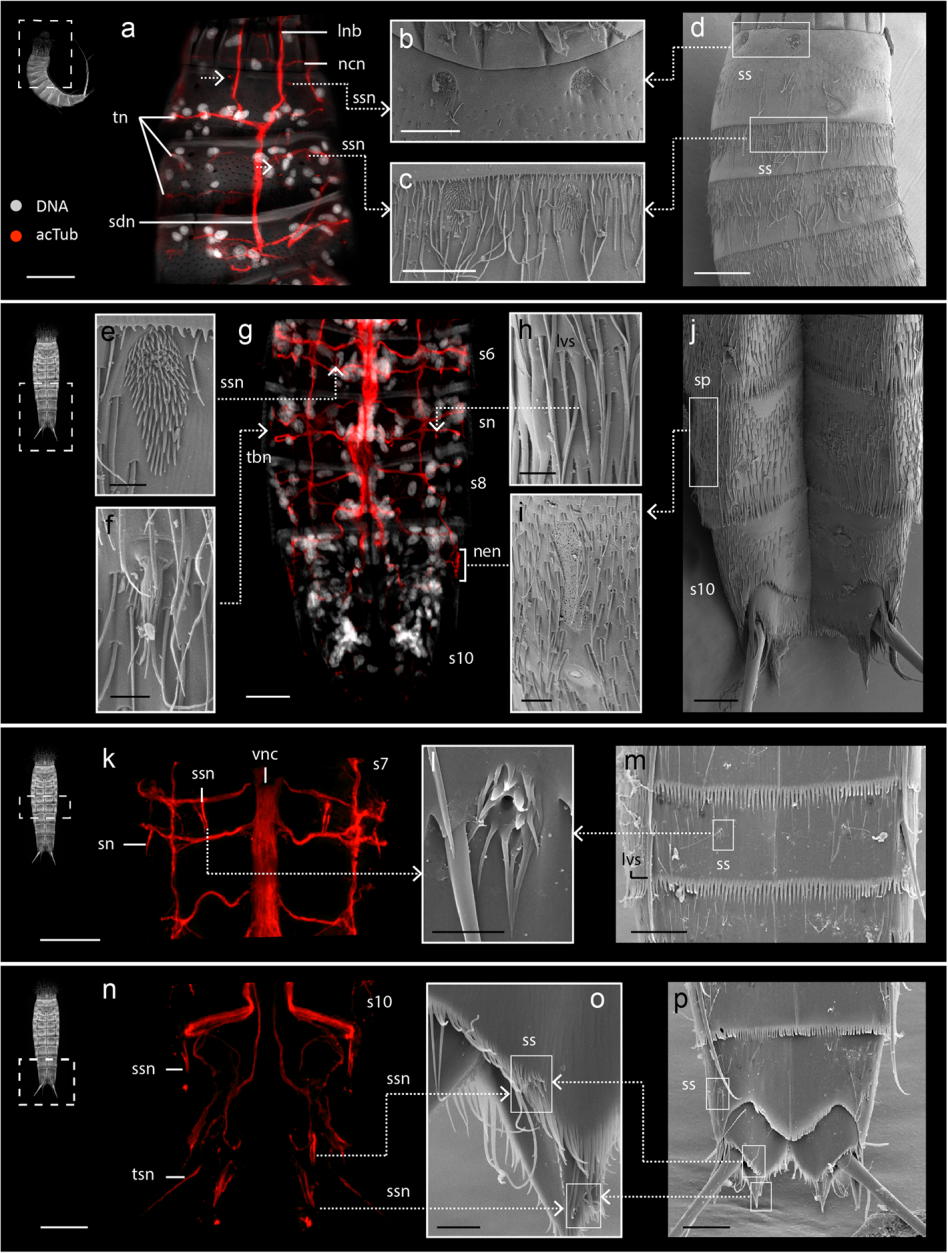
Predicted correlations between internal acetylated α-tubulin-LIR and external cuticular structures in *Echinoderes*. Anterior is up in all panels. Specimen silhouettes with dashed squares indicate the axial region of each z-stack. Color legend applies to all panels. Confocal z-stack projections and SEM micrographs were obtained from different specimens. **a-j** *Echinoderes ohtsukai*. **k-p** *Echinoderes horni*. **a** Confocal z-stack of segments 1–3 in lateral view co-labeled for acTub-LIR (acTub) and DNA. **b** SEM micrograph showing a pair of sensory spots in segment 1 that correspond to the upper boxed area in (d). The upper pair of dashed arrows in (**a**) correspond to innervation of the sensory spots in (**b**). **c** SEM micrograph showing a pair of sensory spots in segment 2 corresponding to the lower boxed area in (**d**). Lower pair of dashed arrows in (**a**) correspond to innervation of the sensory spots in (**c**). **g** confocal z-stack of segments 6–11 in ventral view co-labeled for acTub-LIR and DNA. **e** SEM micrograph of a midventral sensory spot corresponding to innervation (dashed arrow) in (**g**). **f** SEM of a lateroventral fringed tube from segment 7 corresponding to innervation in (**g**). **h** SEM of a lateroventral acicular spine and its innervation in (**g**). **i** SEM of a sieve plate on segment 9 and innervation in (**g**). **j** SEM of segments 8–11 in ventral view showing the position of the sieve plate (boxed area) magnified in (**i**). **k** Confocal z-stack of acTub-LIR within segment 7 in ventral view. **l** SEM of a ventromedial sensory spot and its corresponding innervation in (**k**). **m** SEM of the ventral side of segment 7 showing the relative position (boxed area) of the sensory spot in (**l**). **n** Confocal z-stack of acTub-LIR within segments 9–11 in ventral view. **o** SEM of sensory spots from segment 11 and their correlation with innervation in (**n**). **p** SEM of segments 9–11 in ventral view showing the sensory spots (boxed areas) magnified in (**o**). Scale bars: 20 μm in (**a**, **d**, **n**), 10 μm in (**b**, **c**, **g**, **k**, **j**, **m**, **p**), 2 μm in (**e**, **f**, **h**, **i**, **o**). Abbreviations: lnb, longitudinal neurite bundle; lvs, lateroventral spine; ncn, neck circular neurite; nen, nephridial neurite; s, segment; sdn, subdorsal longitudinal nerve; sn, spine neurite; sp., sieve plate; spn, ss, sensory spot; ssn, sensory spot neurite; tbn, tube neurite; tn, transverse neurite; tsn, terminal spine neurite; vnc, ventral nerve cord. Numbers after abbreviations refer to segment number

**Fig. 5:**
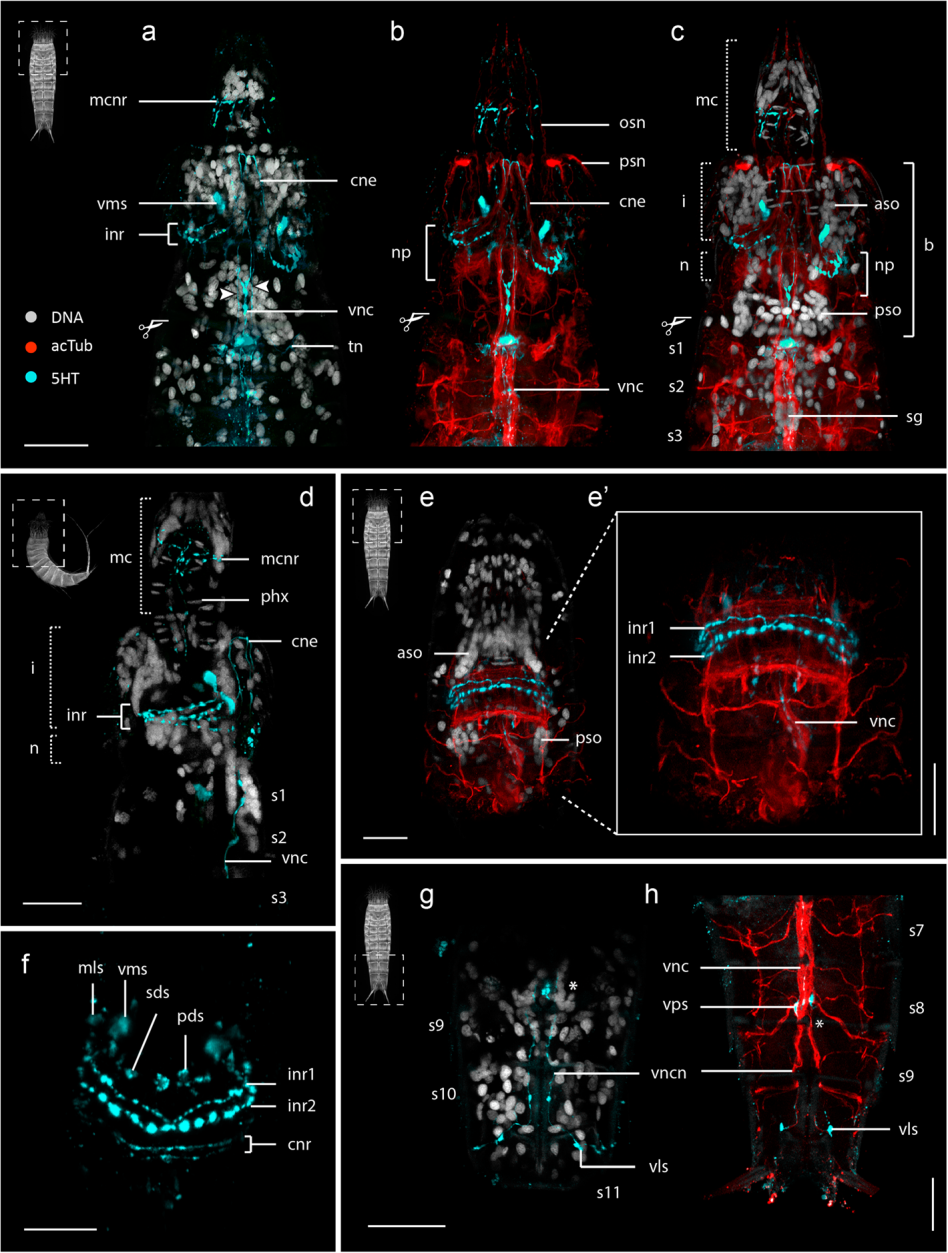
Serotonin-LIR in the nervous system of *Echinoderes*. Confocal z-stack projections co-labeled for 5HT-LIR (**a-h**), acTub-LIR (**b-c, e-e’, h**) and/or DNA (**a, c, d, e, g**). Anterior is up in all panels. Specimen silhouettes with dashed squares indicate the axial region of each corresponding z-stack. Color legend applies to all panels. **a**-**g** *Echinoderes spinifurca*. **h** *Echinoderes horni*. **a-c** Segments 1–3 in ventral views with introvert and mouth cone extended, arrowheads in (**a**) mark the lateral 5HT^+^ strings of the ventral nerve cord. **d** Segments 1–3 in lateral view with introvert and mouth cone extended. **e** Segments 1–4 in dorsal view with introvert and mouth cone retracted. **e’** Magnified view of 5HT-LIR and acTub-LIR within the neuropil region in (**e**). **f** Dorsal view of 5HT-LIR in the neuropil and associated somata. Note the presence of four neural rings and several distinct somata (mls, vms, sds, pds) connected with the first and second rings. **g** Segments 8–11 in ventral view. **h** Segments 7–11 in ventral view. Scissor symbols mark the positions of cuticle dissection to facilitate antibody penetration. An asterisk (**g-h**) marks the location where the ventral nerve cord divides into left and right branches. Scale bars: 20 μm. Abbreviations: aso, anterior somata; b, brain; cne, convergent neurites; cnr, complete neural ring; inr1–2, incomplete neural rings1–2; mc, mouth cone; mcnr, mouth cone nerve ring; mls, 5HT^+^ midlateral somata; i, introvert; n, neck; np, neuropil; osn, oral styles neurites; pds, 5HT^+^ paradorsal somata; phx, pharynx; psn, primary spinoscalids neurite; pso, posterior somata; s, segment; sds, 5HT^+^subdorsal somata; sg, segmental ganglion; tn, transverse neurite; vls, 5HT^+^ ventrolateral somata; vms, 5HT^+^ ventromedial somata; vnc, ventral nerve cord; vncn, ventral nerve cord neurite; vps, 5HT^+^ ventral paired somata. Numbers after abbreviations refer to segment number

**Fig. 6:**
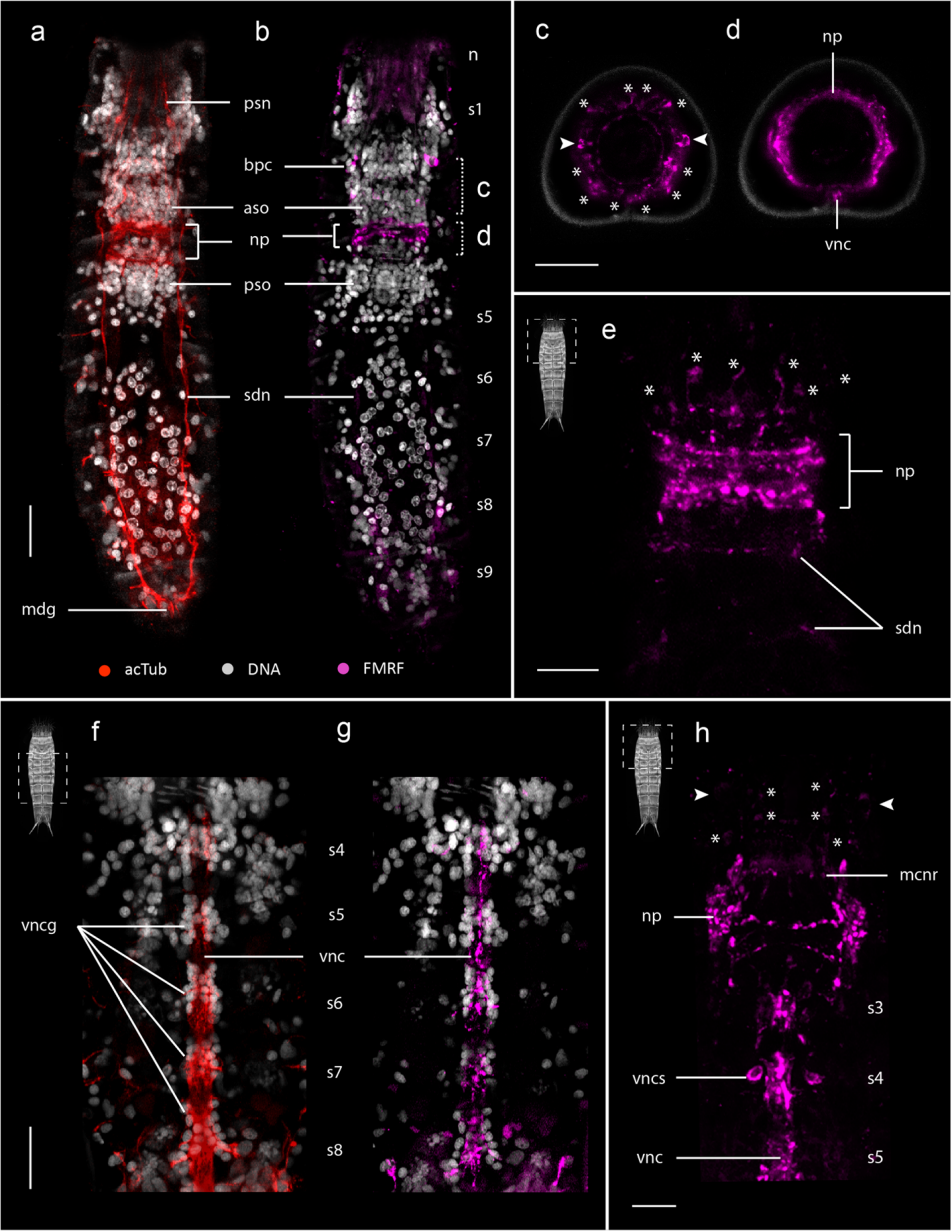
FMRF-LIR in the nervous system of *Echinoderes*. Confocal z-stack projections co-labeled for FMRF-LIR (**b-e**, **g-h**), acTub-LIR (**a, f**) and/or DNA (**a-b**, **f-g**)*.* Anterior is to the top in (**a-b, e-h**); dorsal is to the top in (**c-d**). Specimen silhouettes with dashed squares indicate the axial region of each z-stack. Color legend applies to all panels. **a-d** *Echinoderes spinifurca*. **e-h** *Echinoderes horni*. **a-b** Segments 1–9 in dorsal views with head retracted. FMRF-LIR is detected in the neuropil and several somata associated with the anterior end of the brain and introvert. FMRF-LIR is correlated with acTub-LIR within subdorsal nerves (**a**). **c-d** Anterior views of cross sections through the brain corresponding to axial positions (c and d) in (**b**). **c** multiple FMRF^+^ somata (asterisks, arrowheads) anterior to the neuropil. **d** FMRF^+^ ring-like neuropil and ventral nerve cord. The outer contour in **c** and **d** is cuticular autofluorescence. **e** Dorsal view of FMRF-LIR in the neuropil and associated somata (asterisks). **f-g** Segments 4–8 in ventral view of the same specimen showing acTub-LIR (**f**) and FMRF-LIR (**g**) along the ganglionated ventral nerve cord. **h** Segments 1–4 in ventral view with head retracted showing FMRF-LIR within the neuropil and associated somata (asterisks). Arrowheads mark the position of FMRF^+^ bipolar somata. Note the pair of FMRF^+^ somata (vncs) in segment 4 along the ventral nerve cord. Scale bars, 20 μm in all panels. Abbreviations: aso, anterior somata; bpc, FMRF^+^ bipolar cells; mcnr, mouth cone nerve ring; mdg, middorsal ganglion; np, neuropil; psn, primary spinoscalid neurite; pso, posterior somata; s1–9, segments 1–9; sdn, subdorsal longitudinal nerve; vnc, ventral nerve cord; vncg, ventral nerve cord ganglion; vncs, ventral nerve cord FMRF^+^ somata

**Fig. 7:**
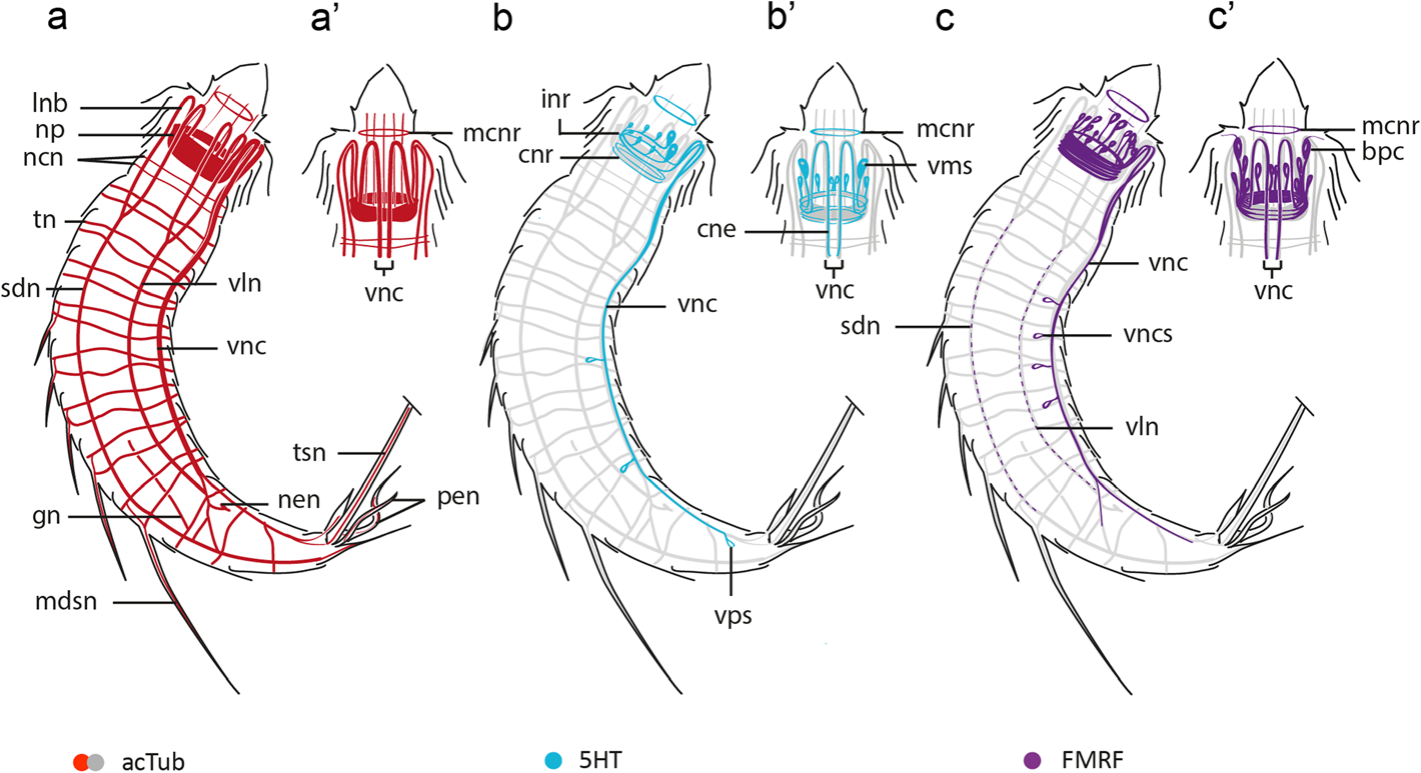
Schematic representations of acetylated α-tubulin-LIR, Serotonin-LIR and FMRF-LIR in *Echinoderes*. **a-c** Lateral overviews with the head extended. **a’-c’** Ventral views of the head. Anterior is to the top in all panels. **b-c’** 5HT-LIR and FMRF-LIR are overlaid upon a background of acTub-LIR (gray). The number of 5HT^+^ and FMRF^+^ somata (vncs) along the ventral nerve cord (**b, c**) varies among specimens and are representative here. Note the approximate orientations and arrangements of 5HT^+^ ventromedial somata (**b’**) and FMRF^+^ bipolar cells (**c’**) relative to neural rings of the neuropil. Abbreviations: bpc, FMRF^+^ bipolar cells; cne, convergent neurites; cnr, complete neural ring; gn, gonad neurite; inr, incomplete neural ring; lnb, longitudinal neurite bundle; mcnr, mouth cone nerve ring; mdsn, middorsal spine neurite; ncn, neck circular neurite; nen, nephridial neurite; np, neuropil; pen, penile spine neurite; sdn, subdorsal longitudinal nerve; tn, transverse neurite; tsn, terminal spine neurite; vln, ventrolateral nerve; vms, 5HT^+^ ventromedial somata; vnc, ventral nerve cord; vncs, ventral nerve cord FMRF^+^ somata

The tubulinergic nervous system in *Echinoderes* reveals a circumpharyngeal brain with several longitudinal neurite bundles originating from the neuropil (np) and extending along the introvert and trunk (Fig. 3). The neuropil includes a condensed set of parallel neurites forming a ring measuring 15–20 μm in diameter (Figs. 2b-c, 3a-c, 5b-c, e-e’, 6a and 7a; Additional file 1), which is constricted on the ventral side (Figs. 2b, 3b and 5b-c). From the anterior end of the neuropil, ten radially arranged longitudinal neurite bundles (lnb) extend anteriorly along the introvert, bend toward the body wall and then extend posteriorly along the trunk (Figs. 2b, 3a-c and 7a). At the level of the first trunk segment, eight of those neurite bundles fuse in pairs to form two subdorsal (sdn) and two ventrolateral nerves (vln) (Figs. 2c-d, 3 and 4a). The ventrolateral nerves extend to segment 9 (Figs. 2a and 3a-b). The subdorsal nerves also extend to segment 9 where they converge into a putative ganglion on the dorsal midline (Figs. 2h, 3a and 6a). From there, two neurites extend from that junction to innervate segments 10–11 (Figs. 2f-h, 3a-b and g). Two additional subdorsal neurites, most likely associated with the gonads (gn) extend posteriorly from within segment 6 and converge with the longitudinal dorsal nerves in segment 9 (Figs. 2h, 3a and 7a).

Of the ten longitudinal tubulinergic neurite bundles that originate from the brain neuropil, the ventromedial pair is the most prominent. This pair of nerves converges between segments 2–3 where it forms part of the ventral nerve cord that extends posteriorly along the trunk. DNA labeling of the ventral nerve cord with propidium iodide shows ganglia that are located medially within segments 1–9 (Fig. 2a-a’ and Fig. 6f). Most cell nuclei of the ventral ganglia are positioned laterally along the cord with longitudinal connectives toward the midline (Fig. 2a’and Fig. 6f). Somata-free connectives extend between ganglia along the ventral cord (Figs. 2a’ and 6f). Within segment 8, the ventral nerve cord diverges posteriorly into left and right bundles (Figs. 2f-g and 3a-b; Additional files 1 and 2). From each branch, peripheral neurites innervate segments 10–11 and extend laterally to make connections with subdorsal neurite bundles (Additional file 2). The subdorsal nerves, ventrolateral nerves and the ventral nerve cord are interconnected through transverse neurites (tn) in a ladder-like pattern within segments 1–9 (Figs. 2a, d, f, g, 3 and 7a; Additional files 1 and 2). The transverse neurites are unpaired in segment 1, paired ventrally in segment 2, and paired circumferentially in segments 3–9. The ventrolateral nerves appear to connect posteriorly with left and right branches of the ventral nerve cord within segment 9, with subdorsal nerves converging medially within segment 9 and then extending posteriorly to innervate the dorsal region of segments 10–11 (Figs. 2h, 3a and 6a; Additional files 1 and 2). The architecture of the tubulinergic nervous system of *Echinoderes* exhibits a segmental organization between trunk segments 1–9.

#### Innervation of cuticular structures

Most of the cuticular structures in *E. horni, E. spinifurca* and *E. ohtsukai* showed acTub-LIR innervation*.* Variation in the numbers and distribution patterns of cuticular structures among species was reflected by variation in their acTub-LIR innervation patterns.

Head region: The most prominent cuticular structures in the head include the pentaradially arranged scalids and the mouth cone styles. A single, relatively thin neurite (spn) extending from the anterior part of the neuropil innervates each scalid (Fig. 2c), with the exception of the first row of scalids (spinoscalids), which are each innervated by at least two neurites (psn) (Fig. 2b-c). The mouth cone comprises four rings of radially arranged oral styles. The first row is the most conspicuous with nine articulated oral styles. From the posterior part of the neuropil, nine radially arranged neurites (osn) extend anteriorly through the neuropil toward the mouth cone. Each of the nine neurites then innervates one of the nine outer oral styles (Figs. 2b, c, 3a-c, 5b and 7a). Each one of these neurites is also connected with one circular neurite (mcnr) at the base of the mouth cone (Figs. 2c, 3a-b and 7a).

Neck region: The neck is innervated by two acTub-LIR circular neurites (ncn), with one located anterior and one located posterior within the ring of placids (Figs. 3a-b, d, 4a and 7a). These circular neurites appear to be connected by short longitudinal neurites; however, the low acTub-LIR did not enable finer resolution of their arrangement within the neck region (Fig. 4a).

Trunk region: Among the three species of *Echinoderes*, there are several cuticular structures (e.g. spines, tubes, sensory spots, sieve plates) on the trunk that exhibit variability in their relative positions and arrangements. In each of the species we examined, spines and tubes were innervated by acTub-LIR neurites extending from transverse segmental neurons within each corresponding segment, with the exception of lateral terminal spines, lateral terminal accessory spines and penile spines of segment 11. These particular spines are innervated by neurites (tsn, ltasn, pen) which emerge from left and right branches of the ventral nerve cord in segments 8–9 (Figs. 2f-h, 3a-b and 7a; Additional files 1 and 2). The sensory spots are oval structures comprised of small papillae and one or two external pores through the surface of the trunk. The external pores of sensory spots appear to be innervated by one or two neurites (ssn) extending from transverse segmental neurons (Fig. 4, Additional file 1). The sieve plates (sp) are paired protonephridial openings through the tergal plates of segment 9; however, there is notable species-specific variability in their position, shape and size. Regardless of whether sieve plates are small and round (*E. spinifurca* and *E. horni*), or large and elongated (*E. ohtsukai*), they are innervated by neurites (spn) emerging from dorsal transverse neurites of segment 9 (Fig. 4g-j, Additional files 1 and 2).

### Serotonergic nervous system

Serotonin-like immunoreactivity (5HT-LIR) was detected within the nervous systems of *E. spinifurca* (17 specimens), *E. horni* (11 specimens) and *E. ohtsukai* (5 specimens). All three species revealed a common pattern of 5HT-LIR in the brain and ventral nerve cord regions of the central nervous system.

Within the brain, we observed four neural rings with 5HT-LIR that were associated with the neuropil (Figs. 5a-f and 7b). The two anterior rings (inr) are conspicuous and incomplete on their ventral side (Fig. 5a, d); the two posterior rings (cnr) are comparatively thin and complete (Fig. 5f). The anteriormost incomplete ring (inr1), is bracelet-shaped and extends dorsally in a semicircular pattern from two relatively large ventromedial somata (vms). Three additional pairs of somata (pds, sds, mls) are also connected with the anteriormost neural ring in paradorsal, subdorsal and midlateral positions, respectively (Figs. 5f and 7b-b’). All of the somata associated with this neural ring are located within the anterior brain region (aso) (Fig. 5a, c, d). We did not detect positive 5HT-LIR somata associated with the second incomplete ring (inr2), which appears to connect with the first incomplete ring (inr1) on its dorsal side (Fig. 5e’, f). The ventral side of inr2 extends and connects with two neurites (cne) that converge with the ventral nerve cord (Figs. 5a-d and 7b-b’). The relative position of these convergent neurites is flexible and depends upon whether the movable head is everted or retracted. The two complete rings (cnr) are less conspicuous, with the emission and detectability of their 5HT-LIR showing variation among individual specimens (Fig. 5f). At the base of the mouth cone, another nerve ring (mcnr) was observed (Figs. 5a, d). We were unable to verify if the mouth cone nerve ring is connected with the incomplete rings of the neuropil.

The relative position of the 5HT-LIR of the ventral nerve cord did not change regardless of the position of the moveable head suggesting that it is fixed within the trunk. The origin of the ventral cord appears to correlate with the ventral convergence of two neurites (cne) that connect with an incomplete neural ring (inr2) that encircles the brain (Fig. 5a, d). From that convergence, paired neurites bifurcate into four observable neurite strings, with the medial pair of strings fusing at the level of the first trunk segment to form a central neurite bundle, and with the lateral pair of strings running parallel to the central bundle along the trunk (Fig. 5a). Along the lateral strings of the ventral cord, there are paired somata (vps) with 5HT-LIR that are arranged in a segmental pattern, as observed within segments 5 and 8 (Figs. 5h and 7b). The central neurite bundle terminates in segment 9. The lateral neurite strings continue into segment 10, where they diverge from the ventral midline and terminate with two ventrolateral somata (vls) showing 5HT-LIR (Fig. 5g, h). Low levels of 5HT-LIR were also correlated with the positon of transverse neurites (tn) in some segments (Fig. 5a). Additionally, 5HT-LIR was detected within the pharyngeal bulb (Fig. 5d); however, labeling of the stomatogastric nervous system was generally incomplete.

### FMRFamidergic nervous system

FMRFamide-like immunoreactivity (FMRF-LIR) was detected in *E. spinifurca* (8 specimens) and *E. horni* (15 specimens). In all specimens, FMRF-LIR was observed in the brain, the ventral nerve cord and several areas corresponding with ventrolateral and subdorsal longitudinal nerves.

Within the brain, FMRF-LIR is localized primarily in the neuropil, and in a combination of at least six or more perikarya and associated neurites within the anterior brain region (Figs. 6b-e, g, h and 7c-c’). FMRF-LIR in the neuropil reveals a broad series of dorsal rings that narrow midventrally along the anterior-posterior axis (Fig. 6h). The associated perikarya extend neurites to dorsal, lateral and ventrolateral locations along the anterior side of the neuropil (Fig. 6c, e, h). All of these anterior perikarya are similar in size, with the exception of a comparatively larger pair of bipolar cells (bpc) that are located at midlateral positions along the brain (Fig. 6b, c, h also indicated with arrowheads). Compared with other cell bodies showing FMRF-LIR, the two bipolar cells are positioned more distally from the neuropil, closer to the base of the introvert scalids, with each cell extending one neurite anteriorly that innervates the first spinoscalids of the introvert (Figs. 6b and 7c-c’). FMRF-LIR was also detected as a thin ring within the basal part of the mouth cone (mcnr) (Figs. 6h and 7c-c’), which appears to correlate with the positions of mouth-cone nerve rings showing acTub-LIR and 5HT-LIR. The two convergent neurites (cne) that extend from the neuropil toward their connections with the ventral nerve cord also showed FMRF-LIR.

The ventral nerve cord was labeled along its length, and is comprised of at least 4–6 FMRF-LIR neurite strings (Fig. 6f-h). A pair of somata (vncs) are located laterally along the ventral nerve cord at least within segments 3, 4, 5 and 6 (Fig. 6g-h). Weak levels of FMRF-LIR were detected in some regions that appear to correspond with positions of ventrolateral longitudinal nerves (vln) and subdorsal longitudinal nerves (sdn) along the trunk (Figs. 6b, e and 7c).

## Discussion

### *Echinoderes* exhibits a conserved neuroanatomy

In agreement with descriptions of variation in external, species-specific cuticular characters across *Echinoderes*, we also detected interspecific variation in the number, position, and presence or absence of peripheral neurites. These peripheral neurites innervate the species-specific cuticular characters within *E. spinifurca*, *E. horni* and *E. ohtsukai* (discussed below). Apart from such specific variation, our immunolabeling experiments generally show that all three species exhibit highly similar tubulinergic, serotoninergic and FMRFamidergic elements within their central and peripheral nervous systems. Thus, these results reinforce our hypothesis of a conserved neuroanatomy in *Echinoderes*. Our findings mostly agree with the ultrastructural studies of Nebelsick [25] in *E. capitatus* regarding the number of nerve cords in the head and trunk, the origin and arrangement of the ventral nerve cord and peripheral innervation of the trunk. However, we also identified transverse neurites connecting dorsal longitudinal nerve cords within each segment, which was probably overlooked in Nebelsick’s description. Regarding innervation of the mouth cone, neck and trunk, our observations also disagree with ultrastructural studies of *E. aquilonius* by Kristensen and Higgins [26]. Innervation of the mouth cone oral styles originates from the posterior end of the neuropil and not from anterior somata of the brain, as previously suggested [26]. Our results further imply that innervation of the neck and the trunk originates from the anterior end of the neuropil instead of from somata in the posterior brain region [26]. Therefore, we suggest that the anterior brain may be involved in the coordination of introvert, neck and trunk functions and the posterior part of the brain may be responsible for controlling the mouth cone and associated stomatogastric functions. We could not find any evidence of ganglia along the dorsal nerve cords as described in *E. aquilonius* [26]. The only dorsal ganglion-like structure we could identify across all three species was consistently located in the middorsal region of segment 9, and possibly involved in the coordination of movement among the posteriormost segments, reception of sensorial stimuli and regulation of the gonads.

Serotonin-LIR was very similar in the neuropil and ventral nerve cord within all three species of *Echinoderes* we examined, and consistent with what was described previously in *E. spinifurca* [29]. However, we identified three additional pairs of neuronal somata associated with the anterior part of the brain that may have a role in coordinating movement of the introvert. We also detected low levels of 5HT-LIR associated with the position of neurites emerging laterally from the ventral nerve cord (e.g. transverse neurites), which could be associated with segmental musculature. FMRF-LIR in *Echinoderes* was consistently detected in the neuropil and associated somata, the mouth cone nerve ring, the ventral nerve cord, and both ventrolateral and subdorsal nerves. This is the first characterization of the FMRFamidergic nervous system in Kinorhyncha; however, due to its widespread immunoreactivity, FMRF amides likely have an important role in the function of the nervous system within *Echinoderes*.

Both 5HT-LIR and FMRF-LIR were detected in neurites of the neuropil and the ventral nerve cord in all three *Echinoderes* species, with both markers co-labeling some elements of the tubulinergic nervous system (Fig. 7). Within the neuropil, FMRF^+^ (FMRF positive) and 5HT^+^ (serotonin positive) somata differ from each other in their relative numbers and positions. Along the ventral nerve cord, four to six FMRF^+^ neurites are located adjacent to three 5HT^+^ neurites, with both sets of markers revealing specific neurites that overlap with acTub^+^ (acetylated α-tubulin positive) neural elements. Pairs of FMRF^+^ and 5HT^+^ somata also are positioned bilaterally to the ventral nerve cord; however, it is not clear whether or not these somata are present in every segment or if the observed 5HT-LIR and FMRF-LIR are associated with the same somata.

#### Innervation of the musculature

*Echinoderes* exhibits segmentally arranged muscles in the trunk, where almost every segment presents paired sets of dorsal, ventral, dorsoventral and diagonal muscle fibers [33]. Nebelsick [25] described some innervation of trunk musculature occurring by extension of muscle-cell processes toward adjacent nerves. We lack ultrastructural evidence in the present study, but based on the conserved neuroanatomy we observed in *Echinoderes*, and taking into account Nebelsick’s ultrastructural results, we could infer that muscles extend toward neurite bundles that are nearest to them, in a similar manner as suggested in *E. capitatus* [25]. Furthermore, segmentally-arranged trunk muscles would be innervated by adjacent transverse neurites that extend from the ventral nerve cord to connect with longitudinal nerves (Fig. 2e, g; Additional files 1 and 2), whereas sets of intersegmental longitudinal muscles along the trunk (see [33]) would be innervated by subdorsal and ventrolateral longitudinal nerves. This is partly in agreement with the work of Kristensen and Higgins [26] where the ventral nerve cord is inferred to exclusively innervate segmental muscles while other longitudinal nerve cords would have both sensory and motor neuron functions.

#### Innervation of the reproductive system

Kinorhynchs are dioecious and possess paired saccate gonads that extend along lateral sides of the trunk from the second or third segment into segment 10 [26, 34]. Our data in *Echinoderes* revealed an extra pair of subdorsal nerves within segments 6–7 (gn) that converge with longitudinal dorsal nerves in segment 9. Based upon their position, we interpret these nerves to be associated with the gonads. Informative descriptions on innervation of the reproductive system in kinorhynchs is scarce. Ultrastructural observations in *E. aquilonius* [26] suggest there is potential innervation of the gonads from ventral ganglia of the last three trunk segments, and innervation of the gonoducts from dorsolateral ganglia of segment 10. We could not find any supporting evidence for this; instead, we found that some of the finer neurites branching out from the ventral nerve cord toward the posteriormost trunk segments may be innervating the gonopores (“gon” in Fig. 3b), which are positioned ventrally at the junction of segments 10 and 11.

#### Innervation of cuticular structures

As mentioned above, the only neuroanatomical differences found in our study among *Echinoderes* are those associated with the innervation of species-specific cuticular characters (sensory spots, spines, tubes), which vary in absence/presence number and position, and the innervation of the nephridiopores (sieve plates). Sensory spots are typically rounded or oval-shaped cuticular chemoreceptive structures with one or two pores surrounded by papillae [24, 26, 34]. The only ultrastructural studies of sensory spots in *Echinoderes* were performed on *E. capitatus* and *E. aquilonius*, which identified two underlying monociliary sensory cells [24, 26]. Our investigation supports these results by showing that each sensory spot is innervated by two fine neurites, most likely corresponding to the axons of each sensory cell (Fig. 4k). However, in all three of the investigated species, these axons emerge from transverse neurites of the trunk, and not from the longitudinal nerves as described by Nebelsick [24].

The trunk spines are needle-like structures with a proximal articulation that have been associated with mechanoreceptive functions [24, 26, 35]. Within each spine there is a duct with sensory endings from underlying sensory cells [36]. Our study clearly shows individual neurites innervating almost the full length of every spine along the trunk (including lateral terminal spines, middorsal spines, lateroventral spines and penile spines). Therefore, immunolabeling supports the sensorial nature of cuticular spines, while detailed ultrastructural data would be required to better resolve the specific functions among the different types of spines.

The cuticular tubes are cylindrical, thin-walled hollow appendages with a distal opening [34, 35]. They are very common across species of *Echinoderes* and exhibit a variety of morphologies. Cuticular tubes have been associated with a secretory function [34, 35, 37]. However, ultrastructural studies in *Echinoderes hispanicus* and *E. cantabricus* did not find unambiguous anatomical associations between cuticular tubes and epidermal glands [36]. Using TEM applications, Nebelsick [38] described bipolar and sensory cells that were associated with particular tubes. Our results show fine individual neurites emerging from transverse neurites along the trunk, and then innervating the basal part of cuticular tubes, in agreement with Nebelsick’s results [38].

The sieve plates are the individual outlet systems for the single pair of protonephridia in *Echinoderes*. The plates can be rounded or oval-shaped and are composed of either multiple perforations or distinct cribriform areas [35]. Sieve plates are always located on the tergal plate of segment 9; however, their exact position and appearance varies among species, and thus serve as a diagnostic character. For example, in *E. ohtsukai* the sieve plates are relatively large (Fig. 4i) when compared with the smaller and more rounded forms in *E. spinifurca* and *E. horni*. Previous ultrastructural investigations of the protonephridia of *E. aquilonius* revealed that they are composed of three biciliary terminal cells, one canal cell, and a non-ciliated nephropore cell that is penetrated by several sensory cells [26]. Our results show that *Echinoderes* nephridiopores seem to be innervated by fine neurites extending from transverse neurites in segment 9, which may correspond with the presence of sensorial cells. *E. ohtsukai* exhibits a larger area of tubulin immunoreactivity underlying the enlarged sieve plate area, suggesting the presence of a higher number of ciliated and sensorial cells than in *E. horni* and *E. spinifurca*, which may be linked with the characteristically larger size of their sieve plates (Fig. 4i).

### Functional aspects of the *Echinoderes* nervous system

*Echinoderes*, as all other kinorhynch genera, has a moveable head with an eversible introvert and a retractable mouth cone [33]. The brain is circumenteric (encircling anterior end of alimentary tract) and is anchored to the epidermis medial to the first row of introvert spinoscalids, and thus spatially integrated with eversion and retraction of the introvert [25, 26, 34]. The ring-shaped brain of kinorhynchs and other scalidophorans may explain the radial pattern of sensory structures around the head and mouth [26, 39]. This particular pattern may have influenced the architectural transition from a radial arrangement of nerves in the head to a bilaterally symmetric arrangement in the trunk [25]. In order to facilitate eversion and retraction of the head, radial somata-free longitudinal nerves are unattached to the body wall in the region of the head [19, 29]. In contrast, the ventral nerve cord appears to be attached to the body wall within the first trunk segment [29]. Segmentally arranged ganglia likely act as anchor points for the ventral nerve cord, whereas somata-free intersegmental neurites (articulation areas) suggest regions of flexibility along the cord. Our observations agree with ultrastructural descriptions of the ventral nerve cord in *E. capitatus* [25]. Furthermore, micrographs of acTub-LIR and FMRF-LIR indicate that intersegmental neurite regions along the ventral cord are sometimes located in a different plane than associated ganglia, and could be misinterpreted as artificially “discontinuous” sections of the cord in ventral views (e.g. Figs. 2a, 5c, 6f-g). This pattern is most obvious during the curvature of the trunk when the ventral cord presents a wave-like appearance. Given the relative rigidity and semi-telescopic arrangement of cuticular segments along the trunk, wave-like flexibility of axon tracts along the ventral nerve cord implies an important functional co-adaptation within a segmented body plan.

### Segmentation in *Echinoderes*

*Echinoderes* is one of the few kinorhynch genera for which internal morphology has been comprehensively investigated. Several studies using TEM, LM and CLSM techniques have demonstrated that species within *Echinoderes* exhibit conserved myoanatomical and neuroanatomical patterns ([29, 33], this paper). Externally, *Echinoderes* exhibits 11 well-differentiated segments that are correlated internally with segmentally arranged components of both muscular and nervous organ systems [25, 29, 33]. Accordingly, segmental morphology in *Echinoderes*, and by extension Kinorhyncha, would be derived developmentally from mesodermal and ectodermal germ-layers, respectively. The arrangement of ventral nerve cord structures, including ganglia, somata-free connectives and peripheral neurons (transverse neurites) appear to follow a typical rope-ladder-like pattern (Fig. 2a-e), although hemiganglia along the cord are not obvious and medial transverse commissures were not unambiguously identified. The head and the neck do not share any segmental characters of the trunk and thus are not considered to be segments [27, 34], in agreement with a lack of segmental organization underlying the nervous system in those regions. Additionally, away from the ventral midline within segments 2–9, ventrolateral and subdorsal longitudinal nerve cords are interconnected by pairs of segmentally iterated transverse neurites that extend from the ventral cord, and continue laterally around the trunk body between those connections. This pattern correlates with segmental organization of the body plan along most of the trunk, and shares some similarity with the orthogonal nervous system described in Priapulida [19]. The above pattern does not persist into the last two segments. Within segments 10–11, longitudinal neurite bundles branch from ventral and subdorsal nerve cords (Fig. 3a-b). Transition in the arrangement of the nervous system in this region is likely associated with multiple cuticular structures located on and within the posteriormost segments, including lateral terminal spines, sexually dimorphic spines (lateral terminal accessory spines in females; penile spines in males), gonopores, tergal extensions and sensory spots. The myoanatomy in segments 10–11 also shows modifications associated with the presence of gonopores, terminal spines, sexually dimorphic spines and tergal extensions [33]. Based upon all of the specialized internal and external morphology that characterizes the posteriormost body region, additional studies along the trunk (e.g. ultrastructure, development, gene expression analyses) could help to resolve outstanding questions on whether neural anatomy is patterned by development of a segmented body in Kinorhyncha, or represents a series of evolutionary transitions from an orthogon [6].

### Comparative neuroanatomy in Kinorhyncha

Prior to our investigation, only two studies of kinorhynch nervous systems using immunohistochemistry and confocal microscopy have been performed, and only with a small number of species: *Echinoderes spinifurca*, *Antygomonas paulae* Sørensen, 2007, *Zelinkaderes brightae* Sørensen, 2007 and *Setaphyes kielensis* (Zelinka, 1928) (summarized in Table 1) [28, 29]. The serotoninergic nervous system was investigated in all these species and tubulin-like immunoreactivity (Tub-LIR) was also characterized in *S. kielensis* (*Pycnophyes kielensis* in Altenburger 2016 [28]). The pattern of Tub-LIR in *S. kielensis* is similar to what we observed in *Echinoderes*, which included a ring-like neuropil, a ganglionated ventral nerve cord composed of two anterior longitudinal neurite bundles that bifurcate in the posterior end, and transverse peripheral neurites emerging from the ventral nerve cord within at least four trunk segments [28]. Our study represents the first successful experiments with FMRF-LIR in Kinorhyncha; therefore, additional studies using this neural marker on other species are needed for a broader comparative framework. Serotonin- \LIR has revealed a similar architecture in the brain and ventral nerve cord in all species studied so far [28, 29]. The observed variation among genera consisted of the number of neuropil rings (2 in *Setaphyes*; 4 in *Antygomonas*, *Echinoderes* and *Zelinkaderes*) and the number of neurons associated with the neuropil [28, 29]. Additionally, the posterior end of the serotonergic ventral nerve cord forms either a ring-like structure in species with a midterminal spine (*Antygomonas* and *Zelinkaderes*) or an inverted Y-like bifurcation in species without a midterminal spine (*Echinoderes* and *Setaphyes*) [29]. As suggested above, there appears to be important correlations between neural organization and species-specific cuticular structures (e.g. terminal spines) situated at the posterior end of kinorhynchs. In the case of 5HT-LIR, we find evidence for potential links between terminal anatomy of the ventral nerve cord and functional morphology of appendage-like characters in mud dragons.

**Table 1:** Comparison of nervous system architecture within Kinorhyncha. Abbreviations: np, neuropil; ios, inner oral styles; oos, outer oral styles; so, somata; ss, sensory spots; vnc, ventral nerve cord. Question marks represent absence of data

Species	Data source	Brain	Number of longitudinal nerves	Transverse neurites (commissures)	Vnc	Innervation of introvert scalids	Innervation mouth cone	Innervation of neck	Innervation of trunk cuticular structures	References
*A. paulae*	CLSM	Circumpharyngeal with 3 regions (So-Np-So)	?	?	Paired origin of vnc	?	Serotonin positive nerve ring	?	?	[29]
*E. capitatus*	TEM	Circumpharyngeal with 3 regions (So-Np-So) Ten lobbed anterior somata	10 fusing into 5 (2 subdorsal, 2 ventrolateral and 1 vnc)	2 per trunk segment	Paired origin of vnc Single ganglion per segment	Neurites from the 10 longitudinal nerves	9 nerves innervating the oos from the “hindbrain” fusing into 5 nerves innervating ios Proximal and terminal nerve ring	10 longitudinal nerves	Spines, tubes (named “setae”) and ss innervated from the 5 longitudinal nerves of the trunk	[24, 25, 38]
*E. aquilonius*	TEM	Circumpharyngeal with 3 regions (So-Np-So)	8	“Circular nerve fibers” in each segment	Paired origin of vnc Double ganglia per segment	Neurites from the anterior perikarya of the brain	10 nerves innervating the oos from the "forebrain" Proximal and terminal nerve ring	Neurites from the longitudinal nerves	Spines, ss, tubes innervated from the middorsal, laterodorsal and ventrolateral cords	[26]
*E. horni* *E. spinifurca* *E. ohtsukai*	CLSM	Circumpharyngeal with 3 regions (So-Np-So) Ten lobbed anterior somata	10 fusing into 5 (2 subdorsal, 2 ventrolateral and 1 vnc)	2 per trunk segment (except for segments 1, 10-11)	Paired origin of vnc Single ganglion per segment	Radially arranged neurites arising from the neuropil	9 nerves innervating the oos arising from the neuropil Anterior nerve ring	10 longitudinal nerves + 2 circular neurites	Spines, ss, tubes, and nephridia innervated from transverse neurites Terminal spines innervated from longitudinal neurites from the vnc	Present study, [29]
*P. greenlandicus*	TEM	Circumpharyngeal with 3 regions (So-Np-So)	8	“Circular nerve fibers” in each segment	Paired origin of vnc Double ganglia per segment	Neurites from the anterior perikarya of the brain	10 nerves innervating the oos from the "forebrain" Proximal and terminal nerve ring	Neurites from the longitudinal nerves	Spines, ss, tubes innervated from the longitudinal cords	[26, 48]
*P. dentatus*	TEM	Circumpharyngeal with 3 regions (So-Np-So)	7	?	Paired origin of vnc (only illustrated)	?	9 nerves innervating the oos from the posterior brain region	?	?	[34, 49]
*S. kielensis*	TEM, CLSM	Circumpharyngeal with 3 regions (So-Np-So)	8 (TEM)	At least one “nerve” per segment (CLSM)	Paired origin of vnc (only illustrated) (TEM) 1 ganglion per segment (TEM)/ 1 ganglion in vnc segment 6 (CLSM)	?	9 nerves innervating the oos (TEM)	?	?	[28, 48]
*Z. brightae*	CLSM	Circumpharyngeal with 3 regions (So-Np-So)	?	?	Paired origin of vnc	?	Serotonin positive nerve ring	?	?	[29]
*Z. floridensis*	TEM	Circumpharyngeal with 3 regions (So-Np-So)	12	?	?	?	9 nerves innervating the oos	?	?	[49]

Ultrastructural studies of kinorhynch nervous systems offer a broad, but incomplete, array of data across genera and species (summarized in Table 1). Most of these studies agree that components of the kinorhynch nervous system consist of a conserved organization of the circumenteric brain (composed of a ring-like neuropil with anterior and posterior concentrations of somata) and several longitudinal connectives within the trunk that are interconnected by segmental pairs of peripheral axon bundles (i.e. transverse neurites) [25–27]. However, the number of longitudinal nerves extending from the brain varies among studies and species (Table 1). Such variability does not reflect the conserved number of nerve cords observed in the present study of *Echinoderes*, in Nebelsick’s ultrastructural study [25], and in unpublished results for additional genera, including *Antygomonas* and *Tubulideres* (Herranz pers. obs.). The disparity between our observations and ultrastructural studies (excluding [25]) may stem from challenges associated with interpreting and/or quantifying structures in TEM micrographs.

Phylogenetic relationships within Kinorhyncha that are based upon comparisons of morphological traits are far from being resolved [40], and therefore neuroanatomical data from additional genera will be important for evaluating hypotheses about the kinorhynch radiation. Based on current evidence, we infer that the ancestral neuroanatomical organization for Kinorhyncha likely included a radial arrangement of 10 longitudinal neurite bundles arising from a circumenteric brain that transition into five longitudinal nerve cords in the trunk. This pattern is based upon the conserved pentaradial symmetry of the head and bilateral symmetry of the trunk that is shared by all kinorhynchs. To date, few molecular phylogenetic studies of Kinorhyncha have been published e.g. [40–43]. After a molecular phylogenetic framework has been established for kinorhynch diversity, traits associated with neural anatomy and other morphological characters (e.g. cuticular structures, patterns of musculature, degree of segmentation) can be mapped onto that framework to more confidently infer the ancestral condition of the nervous system and other organ systems.

### Implications for Scalidophora

**Fig. 8:**
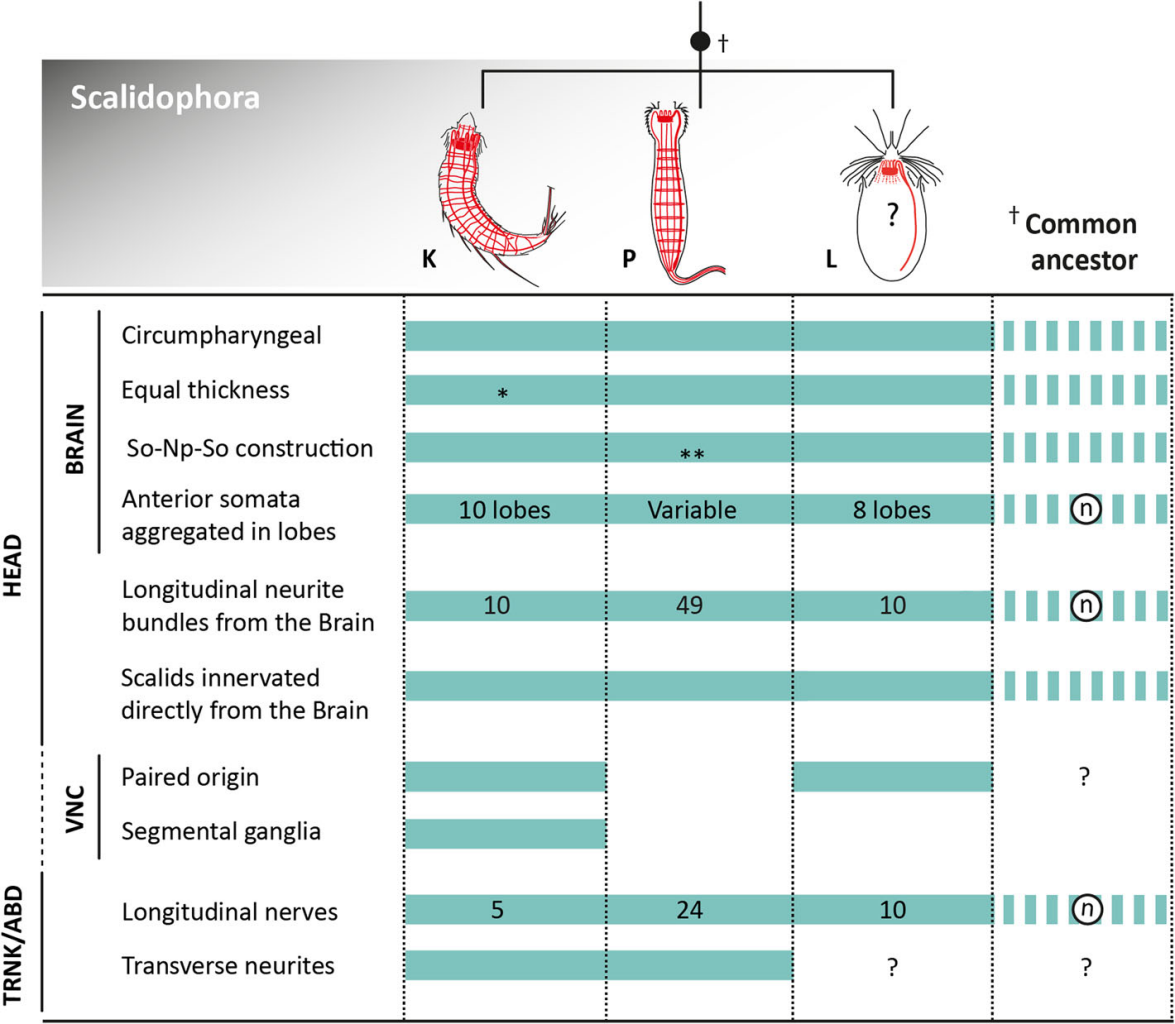
Comparison of nervous system architecture across the Scalidophora. Information presented here is based upon published available data and results from this study (see Discussion). Solid colored bars represent the presence of characters listed on the left side of the figure. Absence of a solid colored bar reflects absence of the associated character. Dashed colored bars represent predicted characters. A question mark reflects uncertainty about predicted character states. Encircled n reflect uncertainty in the organization or number predicted character states. The asterisk in Kinorhyncha indicates that some species show unequal thickness areas along the brain (see Discussion). Two asterisks in Priapulida indicate variability in organization of the brain within the clade. Abbreviations: ABD, abdomen; K, Kinorhyncha; L, Loricifera; Np, neuropil; P, Priapulida; So, somata; TRNK, trunk; VNC, ventral nerve cord

Although robust molecular phylogenetic evidence for Scalidophora is still lacking, comparative morphology clearly shows a unique set of shared characters among loriciferans, kinorhynchs and priapulids (Fig. 8). The primary apomorphic character uniting scalidophorans is a retractable head with an eversible introvert bearing scalids and a protrusible mouth cone [6, 44]. Additional shared characters include radial symmetry of the head and mouth cone appendages, and cuticular flosculi as ciliary sensory receptors [6]. Together with Nematoida (Nematoda and Nematomorpha) [5], scalidophorans also possess a circumpharyngeal brain that is considered a synapomorphy uniting both groups into the Cycloneuralia. However, an absence of molecular support for this larger group suggests that more in-depth morphological studies are required. Within Scalidophora, “cycloneuralian” brain organization varies with respect to the way neuronal somata aggregate within the anterior and posterior sides of the neuropil [2, 21, 22, 26] (Fig. 8). Moreover, in some kinorhynch species the ring-like brain appears to be interrupted on the ventromedial side [29]. We have confirmed that this gap corresponds with the absence of somata in the ventromedial region where the ventral nerve cord emerges from the neuropil (Figs. 5a-d, 7). This pattern most likely serves to accommodate a connection between the ventral nerve cord and the circumpharyngeal brain in *Echinoderes*. Thus, many details of the “cycloneuralian” brain architecture have yet to be discovered and will most likely influence subsequent inferences about evolutionary history within Scalidophora, and between them and closely related ecdysozoans [5, 6].

The transition from a radially symmetric arrangement of nervous system structures in the head (10 longitudinal neurite bundles) to a bilaterally symmetric arrangement of nervous system structures in the trunk (5 longitudinal neurite bundles) has been described in kinorhynchs ([25], present study), but not reported for loriciferans or priapulids [19, 22]. Loriciferans and kinorhynchs each possess 10 longitudinal neurite bundles in the head, with two of them being thicker and forming the ventral nerve cord [23, 25]. However, there is no evidence for the fusion of any of these nerves in either thoracic or abdominal regions in loriciferans. Priapulids show approximately 49 longitudinal neurite bundles that emerge from the brain and extend under and between every row of scalids [45]. Only half of these bundles continue posteriorly into the neck and abdomen, yet there is no evidence of fusion in this taxon either [19].

There is also anatomical variation in the ventral nerve cord within Scalidophora. In all three lineages, the ventral cord emerges anteriorly from the neuropil of the brain and extends posteriorly toward the neck and trunk or abdomen. Within the head regions of kinorhynchs and priapulids the ventral cord is somata-free, presumably to facilitate eversion and retraction of the introvert [19, 29, 45]. It is not clear whether loriciferans share a similar somata-free configuration along the anterior ends of their paired ventral cord, although because of the presence of an introvert and its movement-associated mechanical stress, this seems likely. The question of either a dual or a singular origin of the ventral nerve cord in scalidophorans remains unanswered [19, 21] (Fig. 8). Loriciferans have a paired, ventral longitudinal nerve cord that is fused [22]; this is similar to kinorhynchs, where the ventral cord is also paired and originates as ventral neurite bundles that emerge from the brain and become fused in the trunk region. In contrast, priapulids show a single nerve cord emerging from the neuropil of the brain and extending toward the abdomen [19, 45]. Determination of either ancestral or derived character states for this component of the nervous system is not yet possible because internal relationships within Scalidophora remain unresolved. However, the presence of an unpaired ventral nerve cord in Nematoida has stimulated suggestions of the unpaired condition being ancestral for Cycloneuralia, and a paired ventral cord being apomorphic for loriciferans and kinorhynchs [21]. Additionally, there are segmentally arranged ganglia along the ventral nerve cord in kinorhynchs ([25, 29] and this study). In loriciferans, pharyngeal, thoracic and caudal ganglia have been described along the ventral nerve cord, however there is no evidence of a segmental arrangement, whereas priapulids have no distinct ganglia along the ventral nerve cord [22, 23, 45].

As previously discussed, distinct ganglia might become organized during development of the segmented body plan in kinorhynchs, a pattern that also is absent in all other cycloneuralian taxa. Therefore, since body segmentation and ganglionated ventral cords are both present in other ecdysozoans, but are not observed in loriciferans, priapulids, nematodes or nematomorphs, these traits are inferred to have arisen independently within Ecdysozoa.

Determining which suite of characters are ancestral or derived for Scalidophora is challenging without a robust molecular phylogenetic framework for the group (Fig. 8). Nonetheless, on the basis of available molecular and morphological evidence, we hypothesize that the last common ancestor of Scalidophora would likely possess the following traits: (i) a radially symmetric head with a terminal mouth; (ii) circumpharyngeal brain of equal thickness with an axial pattern of somata-neuropil-somata, including clustered anterior somata; (iii) an anteriorly soma-free, unpaired ventral nerve cord with longitudinal nerves emerging from the brain neuropil that innervate the head, neck and abdomen; and (iv) scalids innervated directly from the brain. In this regard, it is essential that future studies focus their efforts on resolving the internal relationships within Scalidophora, and the Ecdysozoa, with special attention given to the neglected meiofaunal lineages such as kinorhynchs, loriciferans and closely associated groups.

## Conclusions

This study provides the first comprehensive description of the nervous system in *Echinoderes* using confocal laser scanning microscopy (CLSM) and digital three-dimensional (3D) reconstruction. Across all three species investigated here (*E. horni*, *E. spinifurca* and *E. ohtsukai*), the neuroanatomical structure and organization is highly conserved (with the exception of the innervation of cuticular structures). With this level of conservation, we may now extrapolate our results to most, if not all, species of *Echinoderes*, thus extending our collective understanding of the nervous system to more than 40% of the species within Kinorhyncha. Based upon the results of this investigation, nervous system architecture within the adult stages of *Echinoderes* includes the following characteristics: (i) a circumpharyngeal brain with distinct clusters of anterior and posterior somata flanking a centralized neuropil; (ii) individual neurites connecting the anterior neuropil with introvert scalids; (iii) nine neurites connecting the posterior neuropil with the mouth cone outer oral styles; (vi) ten radially arranged neurite bundles that originate from the anterior neuropil and fuse into five radially arranged pairs that extend along the trunk to form two subdorsal longitudinal nerves, two ventrolateral longitudinal nerves and the ventral nerve cord; (v) paired, ganglionated ventral nerve cord within segments 1–9; and (vi) two transverse neurites per segment connecting subdorsal, ventrolateral and ventral longitudinal neurite bundles within segments 2–9.

The nervous system in *Echinoderes* shows a segmented pattern that correlates with the segmental organization present along most of the trunk. Posterior trunk segments exhibit modifications of the nervous system that are likely associated with sensorial, motor or reproductive functions. As in *Echinoderes*, all kinorhynch genera show, to some extent, an externally segmented trunk; however, it is unclear whether external segmentation across Kinorhyncha is always mirrored by internal segmentation of their organ systems such as musculature and the nervous system. External and internal patterns of segmentation are clearly visible in *Echinoderes*. Available comparative data within Kinorhyncha are too scarce to reach a broader conclusion. Therefore, in order to better understand the evolution and extent of segmentation in the only group of segmented cycloneuralians, it will be necessary to comprehensively reconstruct the anatomical systems in additional genera and species of kinorhynchs.

## Methods

### Field sampling

The following research activities were performed in association with the Smithsonian Marine Station at Fort Pierce (SMSFP), Florida, USA, or the University of British Columbia (UBC), Vancouver, BC Canada. Specimens of *Echinoderes spinifurca* and *Echinoderes horni* were collected in September 2012 from three sampling stations offshore of the Fort Pierce Inlet. Sampling stations were located three, four and five nautical miles east of the inlet mouth at 27° 28.33′ N, 80° 13.68′ W, 27° 28.19′ N, 80° 12.76′ W and 27° 30.01′ N, 80° 12.69′ W, respectively. Benthic sediment samples were collected at water depths of 13–15 m with a Higgins anchor dredge on a 0.64 cm cable deployed by hydraulic winch from the RV Sunburst. Specimens of *Echinoderes ohtsukai* were collected in September 2016 from Fanny Bay on the east coast of Vancouver Island, British Columbia at 49°30.071′N, 124°48.611′W. Sampling of *E. ohtsukai* was performed within estuarine conditions (salinities at 20–24‰), where sediments were collected by shovel from the uppermost oxygenated layer of intertidal muds. Kinorhynchs were extracted from all sediment types following the Bubble and Blot technique of Higgins [35, 46]. Live specimens were isolated, identified, and fixed with 4% paraformaldehyde in filtered seawater for 1 h at 4 °C. Following fixation, specimens were washed with multiple exchanges of phosphate buffered saline (PBS 1X) and stored at 4 °C in PBS containing 0.05% sodium azide (NaN_3_) to prevent microbial growth and contamination.

### Scanning electron microscopy (SEM)

Fixed specimens were dehydrated through the following graded series of ethanol dilutions: 20–50–70-90-95-100%. Dehydrated specimens were dried with CO_2_ in a Tousimis Samdri-790 Critical Point Dryer (Tousimis Research Corp., Rockville, MD), mounted on aluminum stubs and sputter coated with gold-palladium. Electron micrographs were produced with a HITACHI S4800 (SMSFP) or a HITACHI S4700 (UBC) field emission scanning electron microscope (SEM; Hitachi High-Technologies America, Pleasanton, CA). Sputter coating and SEM imaging of *E. spinifurca* and *E. horni* were performed at the Horticultural Research Laboratory of the United States Department of Agriculture (USDA) in Fort Pierce, FL. Sputter coating and SEM imaging of *E. ohtsukai* was performed at the Bioimaging Facility at University of British Columbia.

### Immunohistochemistry (IHC) and confocal laser scanning microscopy (CLSM)

Kinorhynch specimens selected for IHC and CLSM experiments consisted of *E. spinifurca* (*n* = 27), *E. horni* (*n* = 35) and *E. ohtsukai* (*n* = 14). Prior to IHC treatments, the cuticles of all specimens were permeabilized by removal of terminal spines or micro-dissection of the terminal segment using a micro scalpel. Dissected animals were incubated in PBT (PBS 1X + 0.5% Triton X-100) for 30 min, followed by incubation in blocking solution (PBT + 0.5% bovine serum albumin + 10% normal goat serum) at 4 °C overnight to inhibit nonspecific binding of antibodies. Specimens were then treated with the following primary antibodies: rabbit anti-serotonin (Sigma-Aldrich, cat# S5545), mouse anti-acetylated α-tubulin (Sigma-Aldrich, cat# T6793) and rabbit anti-FMRFamide (Immunostar, cat# 20091). Different treatments were performed by combining: mouse anti-acetylated α-tubulin with either rabbit anti-serotonin or rabbit anti-FMRFamide. All primary antibody treatments were administered at a concentration of 1:400 in blocking solution at 4 °C for 72 h. Primary antibodies were removed with multiple exchanges of PBT. Specimens were then treated with Alexa-conjugated secondary antibodies: goat anti-rabbit Alexa Fluor 488 F(ab’)2- (Invitrogen, cat# A11070) and donkey-anti-mouse Alexa Fluor 647 (Invitrogen, cat# A31571) at a concentration of 1:400 in blocking solution at 48 °C for 72 h. Fresh preparations of primary and secondary antibody solutions were exchanged each day during a 3-day incubation cycle. Secondary antibodies were removed with multiple exchanges of PBT, followed by three 15-min exchanges of PBS prior to mounting. Subsets of specimens were co-labeled for filamentous actin (F-actin) proteins of the musculature by treatment with Alexa Fluor 633 phalloidin (Invitrogen, cat# A-22284) at 1:100 in PBT, and for DNA within individual cell nuclei by treatment with propidium iodide (PI) (Sigma Aldrich cat# P4170) at 1 μg/mL PBT (final working solution). All incubations were performed in Pyrex spot plates, in the dark while rocking.

For CLSM, specimens were immersed in a solution of glycerol (60% glycerol + PBS 1X) for 30 min, then individually mounted in glycerol on glass slides with a coverslip elevated above each specimen with modeling clay. Specimens were analyzed and imaged with a Zeiss LSM 510 confocal laser scanning microscope (Carl Zeiss, Thornwood, NY) at the SMSFP, or an Olympus FV1000 Multiphoton confocal laser scanning microscope at the UBC Bioimaging facility. Optical sections and z-stack projection micrographs were compiled with Fiji, version 2.00 (Wayne Rasband, National Institutes of Health). For three-dimensional reconstructions z-stacks were surface rendered in Imaris 7.5.0 (Bitplane AG, Zürich, Switzerland). Original SEM and CLSM micrographs were edited (e.g. levels, rotation plane, contrast and brightness) with Adobe Photoshop CS6 (Adobe Systems Incorporated, San Jose, CA) and Fiji. Schematics and figure plates were prepared with Adobe Illustrator CS6 (Adobe Systems Incorporated, San Jose, CA). Positional information used for the identification of external characters, internal anatomy, functional morphology and associated molecular labeling by PI and individual antibody-like immunoreactivity followed the terminology and taxonomic standards for kinorhynchs by Sørensen and Pardos [35] and Herranz et al., [29]. Nervous system terminology follows the neuroanatomical glossary of Richter et al. [47].

## Additional files

Additional file 1: Three-dimensional reconstruction of the tubulinergic nervous system (acetylated α-tubulin-LIR) in *Echinoderes horni.* a-b Segments 1–6 in ventral view, head is retracted. c-d Segments 6–11 in ventral view. e Segments 6–11 in dorsal view. f Segments 6–11 in apical view with dorsal to the top. Musculature (F-actin) is shown in greyscale in (b, d-f). Anterior is to the top in (a-e). Abbreviations: s1–11, trunk segment number. Scale bar, 10 μm. (TIF 4245 kb)

## Abbreviations

aso: Anterior somata
b: Brain
bpc: Bipolar cells
cne: Convergent neurites
cnr: Complete nerve ring
gn: Gonad neurite
gon: Gonopore neurite
h: Head
i: Introvert
inr: Incomplete neural ring
ios: Inner oral styles
lnb: Longitudinal neurite bundle
ltasn: Lateral terminal spine neurite
lts: Lateral terminal spine
lvs: Lateroventral spine
mc: Mouth cone
mcnr: Mouth cone nerve ring
mdg: Middorsal ganglion
mds: Middorsal spine
mdsn: Middorsal spine neurite
mls: Midlateral somata
n: Neck
ncn: Neck circular neurite
nen: Nephridial neurite
np: Neuropil
oos: Outer oral styles
osn: Oral style neurite
pds: Paradorsal somata
pen: Penile spine neurite
phx: Pharynx
psn: Primary spinoscalids neurite
pso: Posterior somata
s: Segment
sdn: Subdorsal longitudinal nerve
sds: Subdorsal somata
sg: Segmental ganglion
sm: Segmental muscle
sn: Spine neurite
sp: Sieve plate
spn: Spinoscalid neurite
ss: Sensory spot
ssn: Sensory spot neurite
tbn: Tube neurite
te: Tergal extension
tn: Transverse neurite
tsm: Terminal spine muscle
tsn: Terminal spine neurite
tu: Tube
vln: Ventrolateral nerve
vls: Ventrolateral somata
vms: Ventromedial somata
vnc: Ventral nerve cord
vncg: Ventral nerve cord ganglion
vncn: Ventral nerve cord neurite
vncs: Ventral nerve cord somata
vps: Ventral paired somata
