# Supplementary figures and images for: Neuroanatomy of mud dragons: a comprehensive view of the nervous system in Echinoderes (Kinorhyncha) by confocal laser scanning microscopy
Source: BMC Evol Biol. 2019 Apr 8;19:86. doi: 10.1186/s12862-019-1405-4 (PMC6454755; doi:10.1186/s12862-019-1405-4)

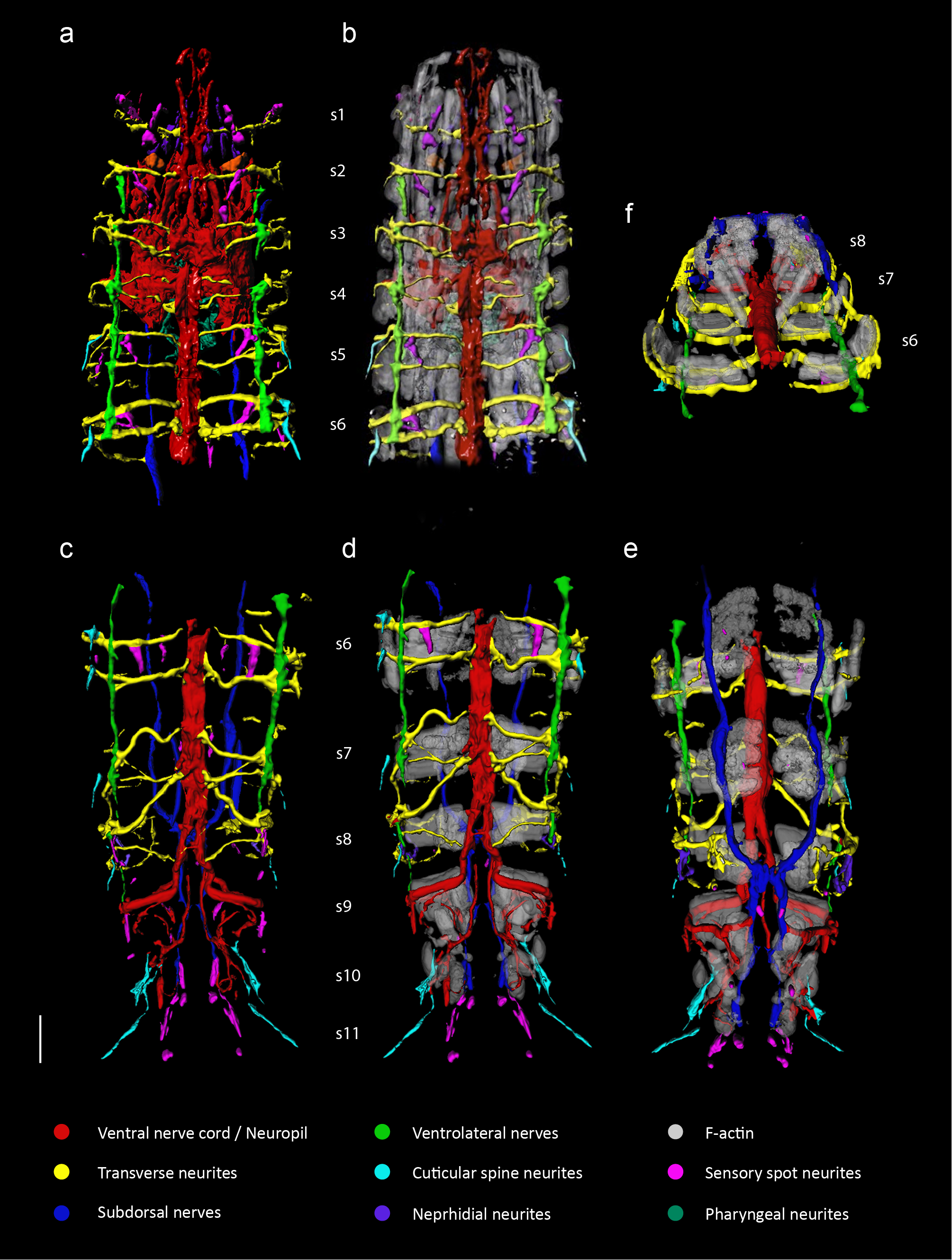

Supplement: Supplementary file 2 — Three-dimensional reconstruction of the tubulinergic nervous system (acetylated α-tubulin-LIR) in Echinoderes horni. a-b Segments 1–6 in ventral view, head is retracted. c-d Segments 6–11 in ventral view. e Segments 6–11 in dorsal view. f Segments 6–11 in apical view with dorsal to the top. Musculature (F-actin) is shown in greyscale in (b, d-f). Anterior is to the top in (a-e). Abbreviations: s1–11, trunk segment number. Scale bar, 10 μm. (TIF 4245 kb) [file 12862_2019_1405_MOESM1_ESM.tif]
